# Is decision-making impairment an endophenotype of Anorexia Nervosa?

**DOI:** 10.1192/j.eurpsy.2022.403

**Published:** 2022-09-01

**Authors:** L. Di Lodovico, M. Lachatre, J. Marcheselli, A. Versini, N. Ramoz, P. Gorwood

**Affiliations:** 1 GHU Paris Psychiatrie, Clinique Des Maladies Mentales Et De L’encéphale, Paris, France; 2 LMD/IPSL, Ecole Polytechnique, Institut Polytechnique De Paris, Ens, Psl Université Sorbonne Université, Cnrs, Palaiseau, France; 3 International School for Advanced Studies (SISSA), Chemistry, Trieste, Italy; 4 Université de Paris, Ipnp, Inserm U1266, Paris, France

**Keywords:** endophenotype, decision making, iowa gambling task, Anorexia nervosa

## Abstract

**Introduction:**

Patients with anorexia nervosa (AN) show impaired decision-making ability, but it is still unclear if this is a trait marker, i.e. a stable endophenotype of AN, or a state parameter, i.e. being explained by present symptoms and associated comorbidity.

**Objectives:**

We aimed to determine whether decision-making impairment is an *endophenotype* of AN. We hypothesized that decision-making alteration would not respect the criteria of an endophenotype, and that these alterations would have a relationship with illness severity.

**Methods:**

Ninety-one patients with acute AN (A-AN), 90 unaffected relatives (UR), 23 patients remitted from AN (R-AN) and 204 healthy controls (HC) underwent the Iowa Gambling Task (IGT) and psychometric assessments. Prospective Valence Learning model (PVL) was employed to distinguish the cognitive dimensions underlying the decision-making process. Performance at the IGT was compared between the four groups and then analysed according to clinical and psychometric variables.

**Results:**

Patients with A-AN scored worse than UR and HC at the IGT (p<.01). PVL-feedback sensitivity parameter was lower in patients with R-AN and A-AN than in the two other groups (p<.01) and PVL-loss aversion parameter was lower in A-AN than in UR and R-AN (p<.01). Decision-making style, in particular learning and loss aversion parameters, accounted for a significant part of variance of psychopathology in patients with AN (p<.01).

**Conclusions:**

Impaired decision-making represents a state-associated, cognitive hallmark of AN. The aggravation of reward modulation along with illness progression may explain the persistence of symptoms despite their consequences on health. Reversal of decision-making impairment should not be limited by inherited vulnerability.

**Disclosure:**

No significant relationships.

